# Dysregulation of junctional adhesion molecule-A via p63/GATA-3 in head and neck squamous cell carcinoma

**DOI:** 10.18632/oncotarget.8432

**Published:** 2016-03-28

**Authors:** Takuya Kakuki, Makoto Kurose, Ken-ichi Takano, Atsushi Kondoh, Kazufumi Obata, Kazuaki Nomura, Ryo Miyata, Yakuto Kaneko, Takumi Konno, Syunta Takahashi, Tsubasa Hatakeyama, Takayuki Kohno, Tetsuo Himi, Takashi Kojima

**Affiliations:** ^1^ Department of Otolaryngology, Sapporo Medical University School of Medicine, Sapporo 060-8556, Japan; ^2^ Department of Cell Science, Research Institute for Frontier Medicine, Sapporo Medical University School of Medicine, Sapporo 060-8556, Japan

**Keywords:** JAM-A, head and neck squamous cell carcinoma, β-catenin, p63, GATA-3

## Abstract

Junctional adhesion molecule-A (JAM-A), which belongs to the IgG superfamily, is a tight junction molecule associated with epithelial and endothelial barrier function. Overexpression of JAM-A is also closely associated with invasion and metastasis of cancers such as breast cancer, lung cancer and pancreatic cancer. However, little is known about the mechanism in overexpression of JAM-A in head and neck squamous cell carcinoma (HNSCC). In the present study, we found high expression of JAM-A at the protein and mRNA levels in HNSCC tissues, including those of the oropharynx, larynx, and hypopharynx, together with high protein expression of β-catenin, p63, ΔNp63 and GATA-3. Furthermore, in ELISA, a significant increase of soluble JAM-A in the sera of HNSCC patients was observed compared to healthy subjects. Knockdown of JAM-A by siRNA inhibited cell proliferation, invasion and migration in the HNSCC cell line Detroit562 *in vitro*. JAM-A expression in Detroit562 was increased via a distinct signal transduction pathway including NF-κB. Expression of JAM-A, β-catenin, p63 and ΔNp63 in Detroit562 was decreased under hypoxia. Knockdown of p63, ΔNp63 or GATA-3 by siRNAs reduced JAM-A expression in Detroit562. In primary cultured HNSCC cells in which CK7, p63, ΔNp63 and GATA-3 were detected, JAM-A expression was decreased by knockdown of p63 or ΔNp63. These results indicate that JAM-A is a biomarker of malignancy in HNSCC and that plasma soluble JAM-A may contribute to serum-based diagnosis of HNSCC. The mechanism of dysregulation of JAM-A via p63/GATA-3 is important in possible molecular targeted therapy for HNSCC.

## INTRODUCTION

Junctional adhesion molecule (JAM)-A is one of immunoglobulin superfamily molecules, which include JAM-B, -C, -4 and JAM-like [1]. JAM-A is expressed at tight junctions of endothelial and epithelial cells where it increases cell-cell adhesion through homophilic interactions [1–3].

Not only loss of tight junction proteins but also overexpression of the proteins is observed in the pathogenesis of cancer [4]. JAM-A is overexpressed in breast, lung and testis cancers [5–7] and is suppressed in endometrial, pancreatic and renal cancers [8–10]. More recently, Tian et al. reported that the overexpression of JAM-A correlates with metastasis and poor prognosis by inducing an epithelial-mesenchymal transition (EMT) via PI3K and MAPK pathways in human nasopharyngeal cancer [11].

The overexpression of JAM-A enhances cell migration via activation of Rap1 and β1-integrin in breast cancer [12, 13]. JAM-A stabiles HER2 expression in breast cancer via PI3K and MAPK pathways, which results in cell proliferation [14]. It also contributes to intestinal epithelial homeostasis by regulating an Akt/β-catenin signalling pathway [15]. Overexpression of β-catenin increases proliferation of head and neck squamous cell carcinoma (HNSCC) cells and induces dedifferentiation of these cells to cells with stem-like features [16].

p63, which is a member of the p53 family and has two distinct isoforms, TAp63 and ΔNp63, plays an important role in proliferation of various epithelial basal cells [17]. ΔNp63 plays a critical role in skin stem cell renewal and is highly expressed in HNSCC [18]. It is known that p63 is upstream of IKKα in epidermal development via GATA-3 [17].

On the other hand, JAM-A is a single transmembrane protein that has an extracellular domain with two Ig-like loops, and the extracellular domain has a potential cleavage site [19]. Circulating soluble JAM-A is associated with inflammation, angiogenesis, hypertension [20], ischemia and atherosclerosis [21]. However, the changes of plasma soluble JAM-A remain unclear during carcinogenesis.

In the present study, we found high expression of JAM-A at the protein and mRNA levels in HNSCC tissues with high protein expression of β-catenin, p63, ΔNp63 and GATA-3. Furthermore, in ELISA, a significant increase of soluble JAM-A in the sera of HNSCC patients was observed compared to healthy subjects. Knockdown of JAM-A inhibited the cell proliferation, invasion and migration of HNSCC *in vitro*. The total protein expression and the surface expression of JAM-A in HNSCC *in vitro* were dysregulated via a distinct signal transduction pathway including NF-κB and p63/GATA-3.

## RESULTS

### Expression and distribution of JAM-A in head and neck squamous cell carcinoma (HNSCC) tissues

**Figure 1:**
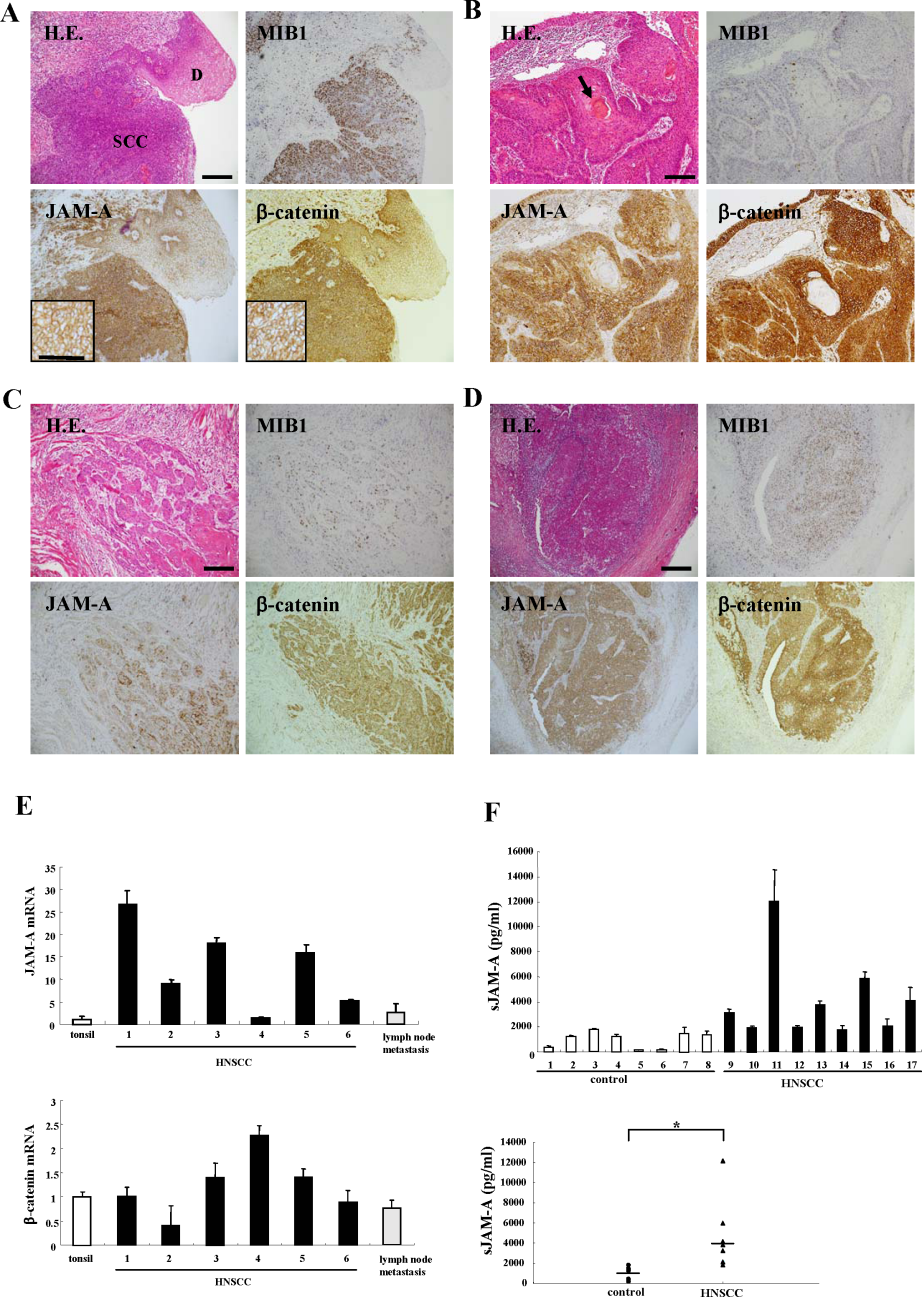
Images of H.E. and immunohistochemical staining of MIB1, JAM-A and β-catenin in tissues of HNSCC patients and dysplastic regions (**A**) HNSCC and dysplasia, (**B**) cancer pearl region, (**C**) invasive region, (**D**) metastatic lymph node. Bar: 100 μm. (**E**) Real-time PCR for mRNAs of JAM-A and β-catenin in tonsil and HNSCC-patient tissues. Results are given as means ± SE. (**F**) ELISA for soluble JAM-A in sera of HNSCC patients and healthy control subjects. Results are given as means ±SE. (**F**) p* < 0.01.

We analyzed the expression and distribution of JAM-A in tissues from HNSCC patients compared to those of β-catenin and MIB1, using immunohistochemistry. JAM-A was predominantly expressed on the membranes of cancer cells in which β-catenin and MIB1 were highly expressed (Figure 1). Higher expression of JAM-A was found in the HNSCC region than in the adjacent dysplastic region (Figure 1A), whereas in the differentiation-induced cancer pearl regions of HNSCC, the level of JAM-A was low as were those of β-catenin and MIB1 (Figure 1B). Furthermore, JAM-A was highly expressed in the invasive region and metastatic lymph nodes (Figure 1C and 1D).

To determine the changes of mRNAs, we performed real-time PCR analysis for JAM-A and β-catenin in six HNSCC tissues and the metastatic lymph nodes compared to three tonsils as a control. The levels of JAM-A mRNA in tissues of five HNSCC patients tissues were 5-25-fold higher than those of the tonsils with the exception of one sample (Figure 1E). The β-catenin mRNA of all HNSCC tissues was less than 2-fold higher than that of the tonsils (Figure 1E). mRNAs of JAM-A and β-catenin were also detected in the metastatic lymph nodes (Figure 1E).

### Expression of plasma soluble JAM-A in serum of HNSCC patients

JAM-A is a single transmembrane protein that has an extracellular domain with two Ig-like loops, and the extracellular domain has a potential cleavage site [19, 18]. We measured plasma soluble JAM-A in the sera of nine HNSCC patients and eight healthy control subjects, using ELISA. The plasma soluble JAM-A levels of some HNSCC patients were markedly increased and the average value for HNSCC patients was significantly higher than in controls (Figure 1F).

### JAM-A knockdown inhibits proliferation, invasion and migration of HNSCC cell line Detroit562

**Figure 2:**
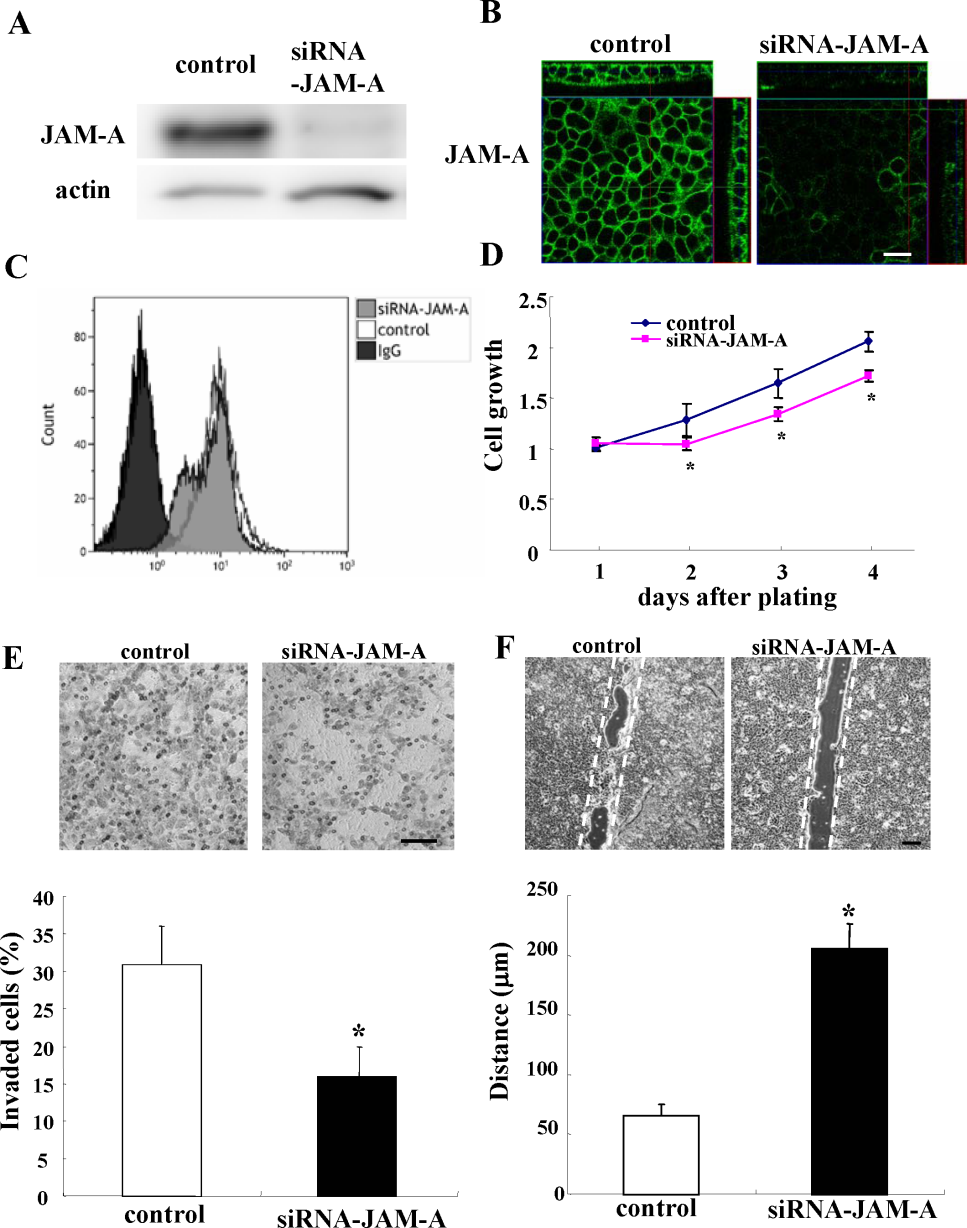
Western blotting (A) immunocytochemical staining (B) and flow cytometry (C) for JAM-A in Detroit562 cells transfected with negative control siRNA or JAM-A siRNA Proliferation assay (**D**) Matrigel invasion assay (**E**) and images of scratch wound assay (**F**) of Detroit562 cells transfected with negative control siRNA or JAM-A siRNA. Bars: 100 μm. The results are shown as bar graphs. (D, E, F) p* < 0.01.

We investigated whether JAM-A knockdown inhibited the proliferation, invasion and migration of HNSCC cell line Detroit562. When the Detroit562 cells were transfected with siRNA of JAM-A, JAM-A expression was decreased in Western blot analysis, immunocytochemical staining and flow cytometry (Figure 2A–2C). In the cell proliferation assay, the growth rate of the cells transfected with siRNA of JAM-A was significantly reduced compared to the control (Figure 2D). In the cell invasion assay, the invading cells were significantly decreased by JAM-A knockdown compared to the control (Figure 2E). In the cell migration assay, JAM-A knockdown significantly decreased wound closure compared to the control (Figure 2F).

### Regulation of expression of JAM-A and β-catenin via a distinct signaling pathway in Detroit562

**Figure 3:**
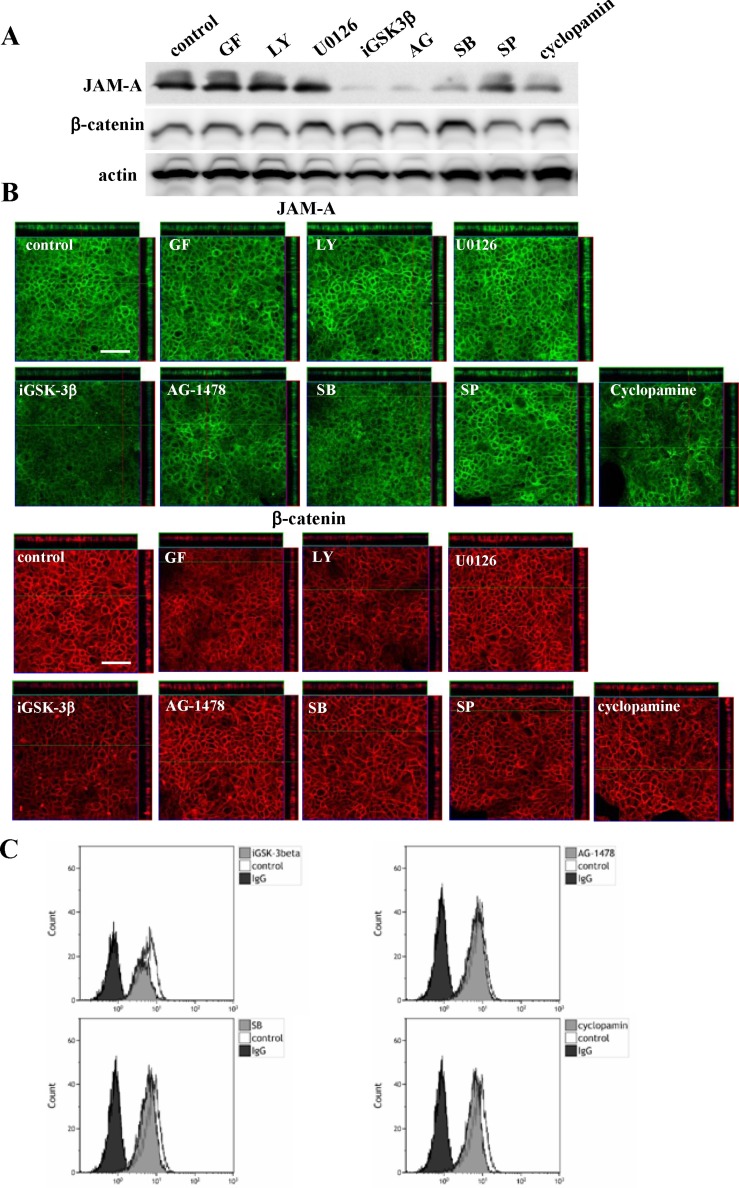
Western blotting (A) and immunocytochemical staining (B) for JAM-A and β-catenin and flow cytometry (C) for JAM-A in Detroit562 cells treated with various inhibitors GF: PKC inhibitor; LY: PI3K inhibitor; U0126: MAPK inhibitor; iGSK-3β: Wnt inhibitor; AG1478: EGFR inhibitor; SB: p38 MAPK inhibitor; SP: JNK inhibitor; cyclopamine: Hedgehog inhibitor. Bars: 100 μm.

To investigate which signal transduction pathways regulated high expression of JAM-A in HNSCC, the Detroit562 cells were treated with various inhibitors of signaling pathways, GF109203X (PKC inhibitor), LY294002 (PI3K inhibitor), U0126 (MAPK inhibitor), iGSK-3β (Wnt inhibitor), AG1478 (EGFR inhibitor), SB203580 (p38 MAPK inhibitor), SP600125 (JNK inhibitor) and cyclopamine (Hedgehog inhibitor). In Western blot analysis, immunocytochemical staining and flow cytometry, the total protein expression level and surface expression level of JAM-A were decreased by iGSK-3β, AG1478, SB203580 and cyclopamine, whereas no change of β-catenin expression was observed (Figure 3A–3C).

**Figure 4:**
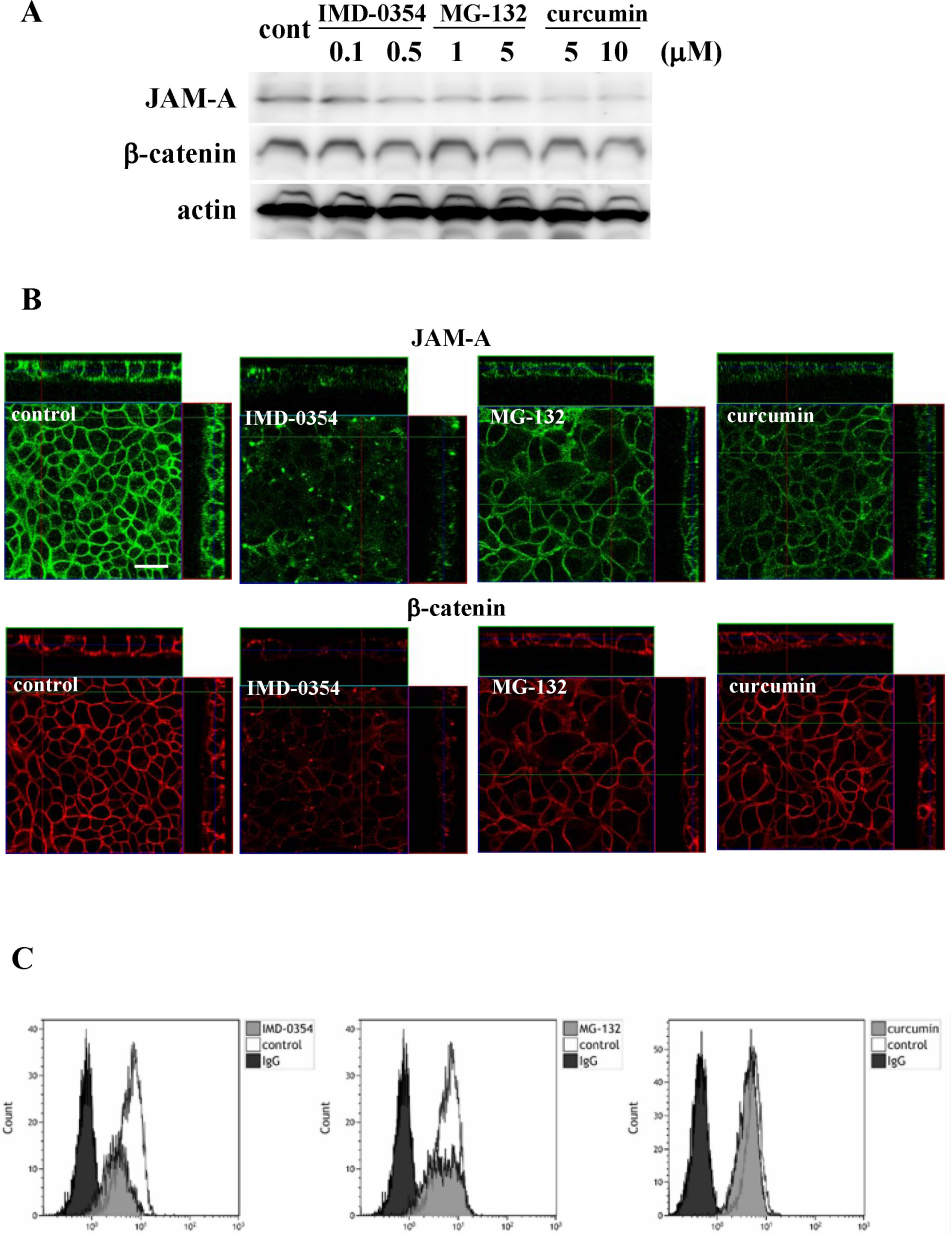
Western blotting (A) and immunocytochemical staining (B) for JAM-A and β-catenin and flow cytometry (C) for JAM-A in Detroit562 cells treated with the NF-κB inhibitors IMD-0354, MG-132 and curcumin. Bars: 100 μm

Furthermore, as it is reported that JAM-A expression is in part regulated via an NF-kB pathway [21], the Detroit562 cells were treated with NF-κB inhibitors IMD-0354, curcumin and MG-132 (proteasome inhibitor). In Western blot analysis, immunocytochemical staining and flow cytometry, total protein expression level and surface expression level of JAM-A were decreased by all NF-κB inhibitors, while no change of β-catenin expression was observed (Figure 4A–4C).

### Expression of p63, ΔNp63 and GATA-3 in HNSCC tissues

**Figure 5:**
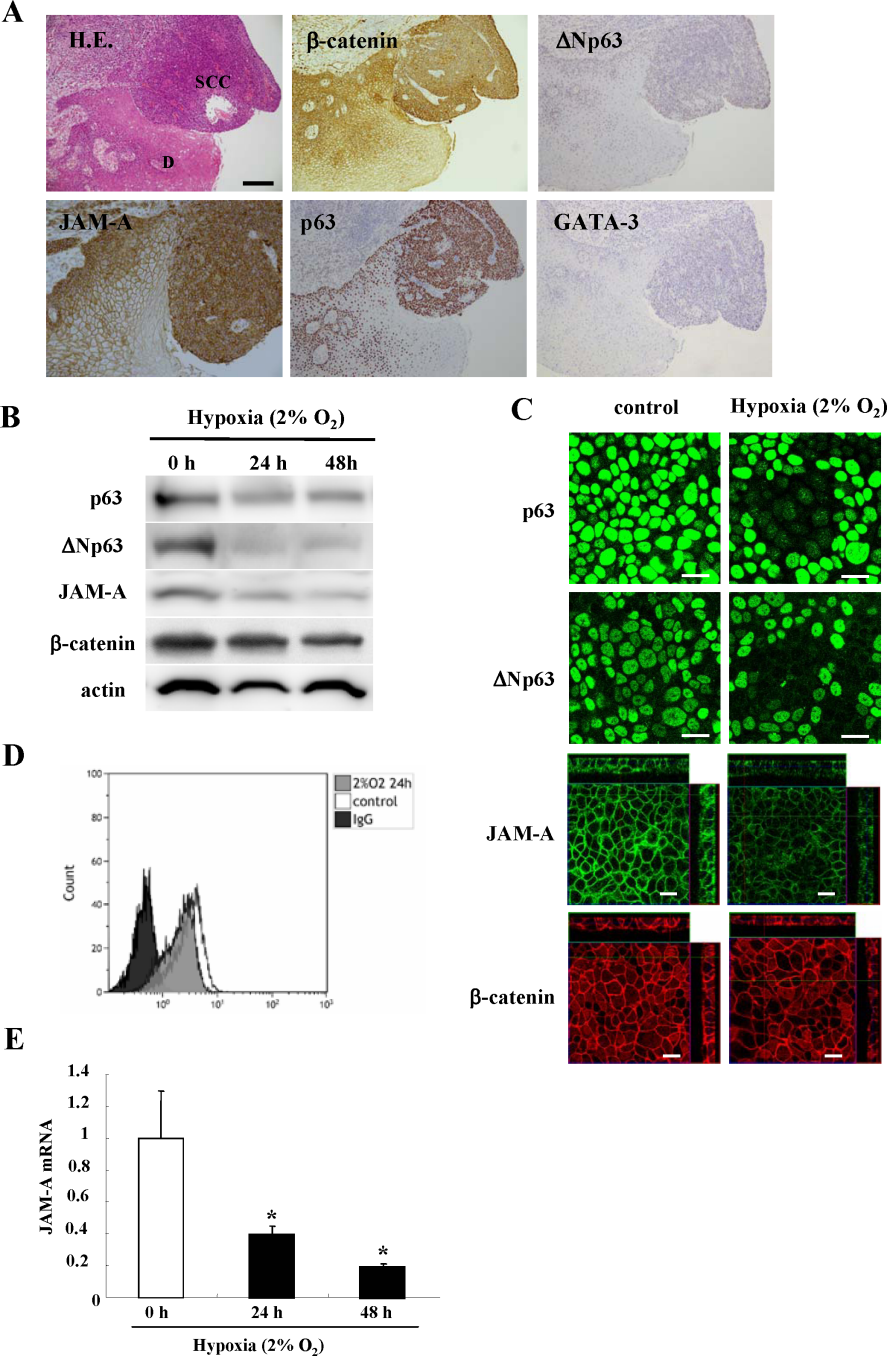
Images of H.E. and immunohistochemical staining (A) of JAM-A, β-catenin, p63, ΔNp63 and GATA-3 in HNSCC and dysplastic regions Bar: 100 μm. Western blotting (**B**) and immunocytochemical staining (**C**) for p63, ΔNp63, JAM-A and β-catenin and flow cytometry (**D**) for JAM-A in Detroit562 cells incubated under hypoxia (2% O_2_). Bars: 20 μm. (**E**) Real-time PCR for JAM-A mRNA in Detroit562 cells incubated under hypoxia (2% O_2_). Results are given as means ± SE. p* < 0.01.

In HNSCC tissues, which highly expressed JAM-A, β-catenin and MIB1, expression of p63, ΔNp63 and GATA-3 was increased compared to the adjacent dysplastic region (Figure 5A).

### Changes in expression of JAM-A, β-catenin, p63 and ΔNp63 in Detroit562 induced by hypoxia

To investigate the relationship between p63 and JAM-A in HNSCC, the Detroit562 cells were incubated under hypoxia (2% O_2_) for 48 h. In Western blot analysis, immunocytochemical staining and flow cytometry, the total protein expression level and surface expression level of JAM-A and β-catenin were decreased under hypoxia in a time-dependent manner (Figure 5B–5D). Furthermore, JAM-A mRNA was also reduced under hypoxia in a time-dependent manner (Figure 5E).

### Regulation of expression of JAM-A and β-catenin via p63, ΔNp63 and GATA-3 in Detroit562

**Figure 6:**
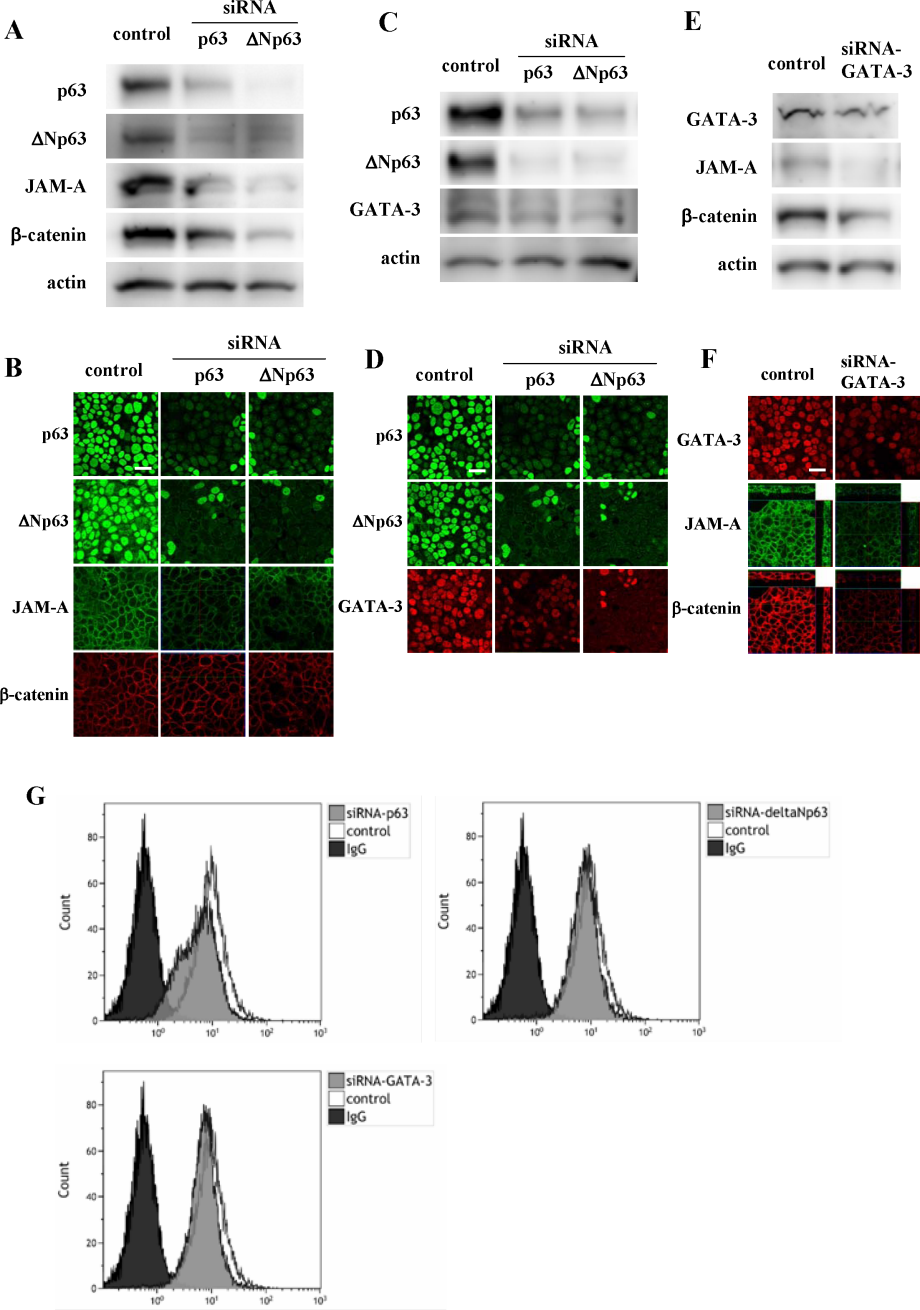
Western blotting (A, C, E) and immunocytochemical staining (B, D, F) for p63, ΔNp63, JAM-A and β-catenin in Detroit562 cells transfected with siRNAs of p63, ΔNp63 and GATA-3 (**G**) Flow cytometry for JAM-A in Detroit562 cells transfected with siRNAs of p63, ΔNp63 and GATA-3. Bars: 20 μm.

p63 affects the development of the epidermis via GATA-3 [17]. To investigate whether expression of p63, ΔNp63 and GATA-3 affected JAM-A expression in HNSCC, the Detroit562 cells were treated with siRNAs of p63, ΔNp63 and GATA-3. When the cells were treated with siRNAs of p63 and ΔNp63, the total protein expression levels and surface expression levels of JAM-A, β-catenin and GATA-3 were decreased in Western blot analysis, immunocytochemical staining and flow cytometry (Figure 6A–6D, 6G). Furthermore, when the cells were treated with siRNA of GATA-3, the total protein expression level and surface expression level of JAM-A and expression of β-catenin and p63 were decreased in Western blot analysis, immunocytochemical staining and flow cytometry (Figure 6E–6G).

### Regulation of expression of JAM-A and β-catenin via p63, ΔNp63 and GATA-3 in primary cultured cancer cells derived from HNSCC

**Figure 7:**
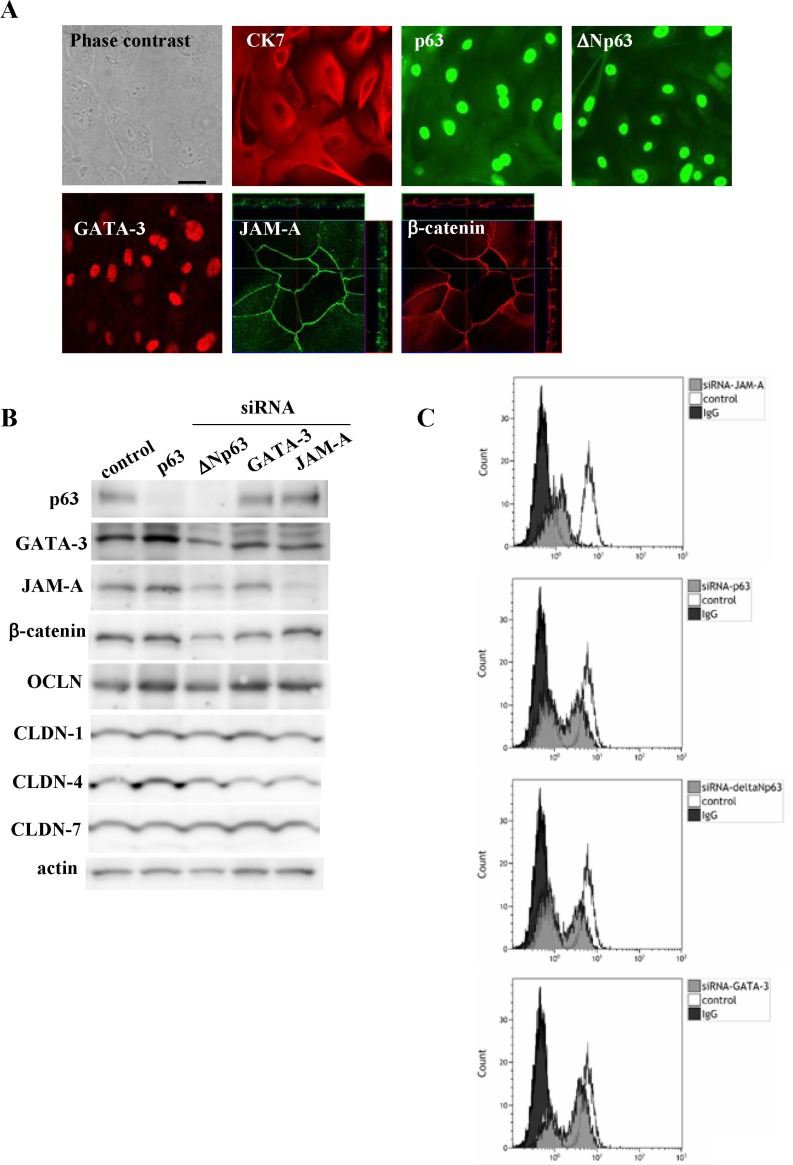
(**A**) Images of H.E. and immunocytochemical staining for CK7, p63, ΔNp63, GATA-3, JAM-A and β-catenin in primary cultured cancer cells derived from HNSCC tissue. Bar: 100 μm. Western blotting (**B**) for p63, ΔNp63, GATA-3, JAM-A, β-catenin, occludin and claudin-1, 4, 7 and flow cytometry (**C**) for JAM-A HNSCC in primary cultured cancer cells transfected with siRNAs of p63, ΔNp63, GATA-3 and JAM-A.

To further confirm that p63, ΔNp63 and GATA-3 affected JAM-A expression in HNSCC, primary cultured cancer cells were derived from HNSCC tissues. In primary cultured HNSCC cells, expression of CK7, p63, ΔNp63, GATA-3, JAM-A and β-catenin was detected by immunocytochemical staining (Figure 7A). The cells were also treated with siRNAs of p63, ΔNp63, GATA-3 and JAM-A. In Western blot analysis, the total protein levels of JAM-A and β-catenin were only reduced by knockdown of ΔNp63, whereas no change of the tight junction proteins occludin, CLDN-1, -4 and -7 was observed (Figure 7B). In flow cytometry, the surface level of JAM-A was decreased by knockdown of p63, ΔNp63, GATA-3 and JAM-A (Figure 7C).

## DISCUSSION

In the present study, overexpression of JAM-A was found at the protein and mRNA levels in HNSCC tissues, where most cancer cells were positive for MIB1 as a proliferation marker. JAM-A was also highly expressed in the invasive region and metastatic lymph nodes, whereas in the differentiation-induced cancer pearl regions of HNSCC, its level was low. Furthermore, JAM-A knockdown by the siRNA inhibited the proliferation, invasion and migration of HNSCC cell line Detroit562. These findings suggested that JAM-A was overexpressed on HNSCC and the disregulation is closely associated with proliferation, invasion and metastasis of the cancer cells, as with breast, lung and testis cancers [5–7, 11].

JAM-A dimerization by the overexpression enhances cell migration via activation of Rap1 and upregulation of β1-integrin in breast cancer [12, 13]. JAM-A stabiles HER2 expression in breast cancer via PI3K and MAPK pathways, which results in cell proliferation [14]. Furthermore, JAM-A regulates epithelial proliferation through Akt/β-catenin signalling in intestinal epithelial cell proliferation in a dimerization-dependent manner [15]. It is possible that JAM-A dimerization by the overexpression in HNSCC may enhance cell migration and proliferation via these mechanisms.

On the other hand, in the present study, we first found by ELISA that a significant increase of soluble JAM-A in the sera of HNSCC patients was observed compared to healthy subjects. Furthermore, when we examined the changes of plasma soluble JAM-A before and after surgical resection of HNSCC, the values of plasma soluble JAM-A in some HNSCC patients after surgical resection significantly reduced compared to the before (data not shown). As the extracellular domain of JAM-A has a potential cleavage site [19], soluble JAM-A is detected in the serum and is associated with inflammation, angiogenesis, hypertension [20], ischemia and atherosclerosis [21]. Although the plasma-soluble JAM-A should be measured in more samples from patients, including those with other diseases, it is possible that the plasma-soluble JAM-A expression in HNSCC patients may contribute to serum-based diagnosis of HNSCC.

JAM-A expression of the normal airway is regulated via a distinct signal transduction pathway including NF-κB [22]. The mechanisms of the overexpression of JAM-A in cancer cells, including the signal transduction pathways, are currently unknown. In the present study, to investigate which signal pathways affect JAM-A expression in HNSCC, Detroit562 cells were treated with inhibitors of various signaling pathways. The inhibitors of Wnt, EGFR, p38 MAPK, Hedgehog and NF-κB reduced the total protein expression level and the surface expression level of JAM-A in the cancer cells. These results suggested that overexpression of JAM-A in HNSCC was also regulated via a distinct signal transduction pathway including Wnt and NF-κB. The changes of JAM-A expression were almost the same as those of β-catenin expression in HNSCC tissues and Deiroit562 cells. JAM-A contributes to intestinal epithelial homeostasis by regulating an Akt/β-catenin signaling pathway [15]. Wnt/β-catenin signaling maintains tumourigenicity of HNSCC [16]. Accordingly, JAM-A expression of HNSCC may be in part regulated via a Wnt/β-catenin signaling pathway.

In the present study, in HNSCC, which highly expressed JAM-A, high expression of β-catenin, p63, ΔNp63 and GATA-3 was also observed. Overexpression of β-catenin increases proliferation of HNSCC cells and induces dedifferentiation of these cells to cells with stem-like features that may be positive for the stem cell marker p63 [16]. Furthermore, ΔNp63 has a critical role in skin stem cell renewal and is highly expressed in HNSCC [18, 17]. ΔNp63 increases the intranuclear accumulation of β-catenin and the signaling in SCC [23]. p63 is also upstream of IKKα via GATA-3 in epidermal development [17].

In the present study, under hypoxia, expression of JAM-A, β-catenin, p63 and ΔNp63 in Detroit562 cells decreased. In the cancer cells, knockdown of p63 or ΔNp63 reduced expression of JAM-A, β-catenin and GATA-3 and knockdown of GATA-3 reduced JAM-A expression. In primary cultured HNSCC cells, knockdown of p63 or ΔNp63 also reduced JAM-A expression. Furthermore, treatment with the NF-κB inhibitors IMD-0354, curcumin and MG-132 reduced not only JAM-A expression but also p63 expression in Detroit562 cells (Supplementary Figure 1).

Our findings indicate that JAM-A is a malignant biomarker of HNSCC and that plasma soluble JAM-A may contribute to the serum-based diagnosis of HNSCC. The mechanism of the dysregulation of JAM-A via p63/GATA-3 is important in possible molecular targeted therapy for HNSCC.

## MATERIALS AND METHODS

### Reagents and antibodies

Inhibitors of PKC (GF109203X), PI3K (LY294002), MAPK (U0126), p38 MAPK (SB203580) and Epidermal growth factor (EGF) receptor (AG1478) were purchased from Calbiochem-Novabiochem Corporation (San Diego, CA). Inhibitors of JNK (SP600125), NF-κB (IMD-0354), Wnt (iGSK3β) and Hedgehog (cyclopamine) were purchased from Sigma-Aldrich (St. Louis, MO). Curcumin was purchased from Cayman Chemical Corporation (Ann Arbor, MI). A proteasome inhibitor (MG132) was purchased from Calbiochem Novabiochem Corporation (San Diego, CA). Mouse monoclonal anti-MIB1 (Ki-67) and mouse monoclonal anti-p63 (Clone DAK-p63) antibodies were obtained from DAKO (Tokyo, Japan). A rabbit polyclonal anti-ΔNp63 antibody was obtained from BioLegend (Tokyo, Japan). A mouse monoclonal anti-GATA-3 antibody was obtained from Santa Cruz Biotechnology (Texas, USA). A mouse monoclonal anti-cytokeratin7 antibody was obtained from Sigma Aldrich. A mouse monoclonal anti-JAM-A antibody (F11R, 2E3-1C8) was obtained from Abnova (Heidelberg, Germany). A rabbit polyclonal anti-JAM-A antibody was obtained from Zymed Laboratories (San Francisco, CA). A mouse monoclonal anti-β-catenin antibody was obtained from BD Transduction Laboratories (San Jose, CA). Rabbit polyclonal anti-claudin-1, -4, and -7, anti-occludin, and anti-tricellulin antibodies were obtained from Zymed Laboratories (San Francisco, CA). An FITC-conjugated anti-JAM-A antibody was obtained from Hycult biotech (Plymouth Meeting, PA). Alexa 488 (green)-conjugated anti-rabbit IgG and Alexa 594 (red)-conjugated anti-mouse IgG antibodies were purchased from Molecular Probes, Inc. (Eugene, OR).

### Human tissues

As a normal control, three human palatine tonsils were obtained from patients with tonsillar hypertrophy who underwent tonsillectomy at Sapporo Medical University. Twenty head and neck cancer tissues were obtained by tumor resection surgery at Sapporo Medical University. Informed consent was obtained from all patients and this study was approved by the ethics committee of Sapporo Medical University.

### Isolation and culture of human head and neck cancer cells

Human head and neck cancer tissues were minced into pieces 2–3 mm^3^ in volume and washed with phosphate-buffered saline (PBS) containing antibiotics four times. These tissue specimens were suspended in 10 ml of dispersing solution with 0.5 μg/ml DNase I (Sigma) and 0.08 mg/ml Liberase Blenzyme (Roche, Basel, Switzerland) in PBS and then incubated at 37°C for 20 min. The dissociated specimens were subsequently filtrated with 300 μm mesh followed by filtration with 40 μm mesh. After centrifugation at 1200 × g for 3 min, the cells were cultured in serum-free bronchial epithelial growth medium (BEBM, Clonetics Corp. San Diego, CA) supplemented with 0.5 μg/ml hydrocortisone, 5 μg/ml insulin, 10 μg/ml transferrin, 0.5 μg/ml epinephrine, 6.5 μg/ml triiodothyronine, 50 μg/ml gentamycin, 50 μg/ml amphotericin B, 0.1 ng/ml retinoic acid, 0.5 ng/ml epidermal growth factor (Lonza Walkersville, Inc.), bovine pituitary extract (1% vol/vol, Pel-Freez Biologicals, Rogers, AR), 100 U/ml penicillin and 100 μg/ml streptomycin (Sigma-Aldrich). The primary cultured cells were plated on 60-mm culture dishes (Corning Glass Works, Corning, NY, USA), which were coated with rat tail collagen (500 μg dried tendon/ml 0.1% acetic acid) in a humidified, 5% CO_2_: 95% air incubator at 37°C.

### Cultures of cell lines and treatment

The pharynx carcinoma cell line Detroit562 (CCL138) was purchased from ATCC (Manassas, VA) and cultured in minimum essential medium (Sigma-Aldrich) supplemented with 10% fetal bovine serum (FBS, Invitrogen; Carlsbad, CA), 100 U/ml penicillin, 100 μg/ml streptomycin and 50 μg/ml amphotericin B. The cells were plated on 60-mm culture dishes (Corning Glass Works, Corning, NY), which were coated with rat tail collagen (500 μg dried tendon/ml 0.1% acetic acid) in a humidified, 5% CO_2_: 95% air incubator at 37°C. The cells were treated with the inhibitors of signal tranduction pathways, each at 10 μM, for 24 h. Furthermore, the cells were incubated under hypoxia (2% O2) for 48 h.

### RNA interference and transfection

siRNA duplex oligonucleotides against p63 and JAM-A were synthesized by Santa Cruz Biotechnology (Texas, USA). siRNA duplex oligonucleotides against ΔNp63 and GATA-3 were synthesized by Invitrogen (Carlsbad, CA). The sequences were as follows: siRNA of p63 (sense: 5′-GGAAUGACUUCAACUUUGA-3′; antisense: 5′-UCAAAGUUGAAGUCAUUCC-3′), siRNA of JAM-A (sense 5′-UUCGAGUAAGAAGG UGAUUUATT-3′), siRNA of ΔNp63 (sense: 5′-ACAAUGCCCAGACUCAAUU-3′; antisense: 5′-AAUUGAGUCUGGGCAUUGU-3′), siRNA of GATA-3 (sense: 5′-GAGAAA GAGUGCCUCAAGU-3′; antisense: 5′-ACUUGAGGCACUCUUUCUC-3′). Detroit 562 cells and primary cancer cells were transfected with siRNAs of p63, ΔNp63, GATA-3 and JAM-A using Lipofectamine^™^ RNAiMAX Reagent (Invitrogen) at 24 h after plating.

### Immunohistochemical analysis

This study was approved by the ethics committee of the Sapporo Medical University School of Medicine. Human palatine tonsils and head and neck cancer tissues were embedded in paraffin after fixation with 10% formalin in PBS. Briefly, 5-μm-thick sections were dewaxed in xylene, rehydrated in ethanol, and heated with Vision BioSystems Bond Max using ER2 solution (Leica) in an autoclave for antigen retrieval. Endogenous peroxidase was blocked by incubation with 3% hydrogen peroxide in methanol for 10 min. The tissue sections were then washed twice with Tris-buffered saline (TBS) and preblocked with Block Ace for 1 h. After washing with TBS, the sections were incubated with anti-MIB1 (1:20), anti-JAM-A (1:1000), anti-β-catenin (1:400) and anti-p63 (1:300), anti-ΔNp63 (1:400), anti-GATA3 (1:400) antibodies for 1 h. The sections were then washed three times in TBS and incubated with Vision BioSystems Bond Polymer Refine Detection kit DS9800. After three washes in TBS, a diamino-benzidine tetrahydrochloride working solution was applied. Finally, the sections were counterstained with hematoxylin.

### RNA isolation and real-time PCR analysis

Total RNA was extracted and purified using TRIzol (Invitrogen, Carlsbad, CA). One microgram of total RNA was reverse-transcribed into cDNA using a mixture of oligo (dT) and Superscript II reverse transcriptase according to the manufacturer's recommendations (Invitrogen). Real-time PCR detection was performed using a TaqMan Gene Expression Assay kit with a StepOnePlus^™^ real-time PCR system (Applied Biosystems, Foster City, CA). The amount of 18S ribosomal RNA (rRNA) (Hs99999901) mRNA in each sample was used to standardize the quantity of the mRNAs of JAM-A (Hs00170991) and β-catenin (Hs00258305). The relative mRNA-expression levels between the control and treated samples were calculated by the difference of the threshold cycle (comparative C_T_ [ΔΔC_T_] method) and presented as the average of triplicate experiments with a 95% confidence interval.

### Western blot analysis

The cultured cells were scraped from a 60 mm dish containing 300 μl of buffer (1 mM NaHCO^3^ and 2 mM phenylmethylsulfonyl fluoride), collected in microcentrifuge tubes, and then sonicated for 10s. The protein concentrations of the samples were determined using a BCA protein assay regent kit (Pierce Chemical Co.; Rockford, IL, USA). Aliquots of 15 μl of protein/lane for each sample were separated by electrophoresis in 5–20% SDS polyacrylamide gels (Wako, Osaka, Japan), and electrophoretic transfer to a nitrocellulose membrane (Immobilon; Millipore Co.; Bedford, UK) was performed. The membrane was saturated for 30 min at room temperature with blocking buffer (25 mM Tris, pH 8.0, 125 mM NaCl, 0.1% Tween 20, and 4% skim milk) and incubated with polyclonal rabbit anti-p63 (1:1000), anti-ΔNp63 (1:1000), anti-GATA-3 (1:1000), anti-JAM-A (1:1000), anti-β-catenin (1:1000), anti-occludin (1:1000), anti-tricellulin (1:1000), anti-claudin-1, -4, -7 (1:1000) and anti-actin (1:1000) antibodies at room temperature for 1 h. Then the membrane was incubated with HRP-conjugated anti-mouse and anti-rabbit IgG antibodies at room temperature for 1 h. The immunoreactive bands were detected using an ECL Western blot system.

### Immunocytochemical staining

The cultured cells in 35-mm glass-coated wells (Iwaki, Chiba, Japan) were fixed with cold acetone and ethanol (1:1) at −20°C for 10 min. After rinsing in PBS, the cells were incubated with anti-cytokeratin7 (1:200), anti-p63 (1:100), anti ΔNp63 (1:100), anti-GATA-3 (1:100), anti-JAM-A (1:100) and anti-β-catenin (1:100) antibodies at room temperature for 1 h. Alexa Fluor 488 (green)-conjugated anti-rabbit IgG and Alexa Fluor 594 (red)-conjugated anti-mouse IgG (Invitrogen) were used as secondary antibodies. The specimens were examined using an epifluorescence microscope (Olympus, Tokyo, Japan) and a confocal laser scanning microscope (LSM5; Carl Zeiss, Jena, Germany).

### Flow cytometry

For analysis of cell surface JAM-A, Detroit 562 cells and the primary cultured cancer cells were detached from the 60 mm dish by using 1 mM EDTA+0.05% trypsin to prepare 2 × 10^6^ cells. After washing with PBS, the cells were incubated with an FITC-conjugated anti-JAM-A antibody or FITC-mouse IgG (BD Biosciences; Franklin Lakes, NJ) for 30 min at 4°C. Then they were washed with PBS and subjected to analysis with a FACScan flow cytometer (10,000 events/sample; BD Biosciences). All data shown were obtained with gates set on living cells and analyzed with Cell Quest software (BD Biosciences).

### Enzyme-linked immunosorbent assay (ELISA)

The concentrations of soluble JAM-A (sJAM-A) in the sera of the controls and the HNSCC patients were measured using an ELISA kit for human JAM-A (R&D Systems, Minneapolis, MN) according to the manufacturer's instructions.

### Proliferation assay

The cells were seeded onto 96 well culture plates (Corning, NY, USA). Each day, the absorbance of three wells was measured using a Cell Counting Kit-8 (Wako, Osaka, Japan) according to the manufacturer's instructions. The absorption at 450 nm was measured by using an iMark Microplate Reader (Bio-Rad, Hercules, CA).

### Matrigel invasion assay

For invasion assay, we used Matrigel (Becton Dickinson Labware, Bedford, MA) and Cell Culture Insert (pore size 8 μm; Becton Dickinson Labware). Detroit 562 cells were plated onto the upper chamber of a Transwell coated with Matrigel and the lower chamber was filled with human fibroblast conditioned medium containing 10 nM EGF as an adhesive subtrate. Next, the cells were incubated for 24 h. Then the upper chamber was fixed with 100% methanol for 10 min and stained with Giemsa for 20 min. The areas of invading cells were measured by using a microscope imaging system (Olympus, Tokyo, Japan).

### Migration assay

After Detroit562 cells were plated onto 35 mm dishes, they were cultured to confluence. At 24 h after we wounded the cell layer using a plastic pipette tip (P200), we measured the distance of the wound using a microscope imaging system (Olympus, Tokyo, Japan).

### Data analysis

Each set of results shown is representative of at least three separate experiments. Results are given as means ± SEM. Differences between groups were tested by analysis of variance followed by a post hoc test and an unpaired two-tailed Student's *t* test.

## SUPPLEMENTARY MATERIALS FIGURE


