# Supplementary material for: Dysregulation of junctional adhesion molecule-A via p63/GATA-3 in head and neck squamous cell carcinoma
Source: Oncotarget. 2016 Mar 28;7(23):33887–900. doi: 10.18632/oncotarget.8432 (PMC5085126; doi:10.18632/oncotarget.8432)
Supplement: Supplementary file 1 [file oncotarget-07-33887-s001.pdf]

Dysregulation of junctional adhesion molecule-A via p63/GATA-3 in head and neck squamous cell carcinoma

Supplementary Materials

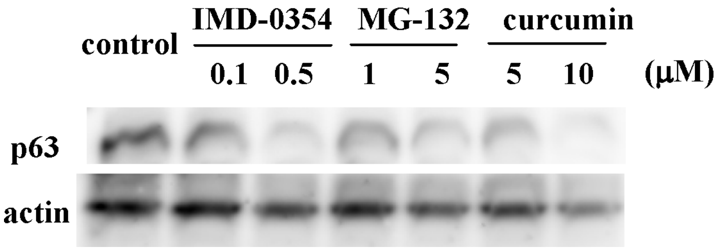

Supplementary Figure S1: Western blotting for p63 in Detroit562 cells treated with the NF-κB inhibitors IMD-0354, MG-132 and curcumin.
